# Novel Prognostic Factors Associated with Cell Cycle Control in Sporadic Medullary Thyroid Cancer Patients

**DOI:** 10.1155/2019/9421079

**Published:** 2019-02-18

**Authors:** Raffaele Pezzani, Loris Bertazza, Elisabetta Cavedon, Simona Censi, Jacopo Manso, Sara Watutantrige-Fernando, Gianmaria Pennelli, Francesca Galuppini, Susi Barollo, Caterina Mian

**Affiliations:** ^1^Endocrinology Unit, Department of Medicine (DIMED), University of Padova, Via Ospedale 105, Padova 35128, Italy; ^2^Associazione Italiana per la Ricerca Oncologica di Base (AIROB), Padova, Italy; ^3^Surgical Pathology and Cytopathology Unit, Department of Medicine (DIMED), University of Padova, Via Gabelli 61, 35121 Padova, Italy

## Abstract

**Background:**

Medullary thyroid cancer (MTC) is a rare neuroendocrine-derived malignancy. It is represented by sporadic and familiar forms, and both can have *RET* oncogene mutations. Numerous markers can be used to define MTC; however, none is generally approved for predicting the outcome of sporadic MTC.

**Aim:**

The aim of this work was to analyze PTTG1/securin and Aurora kinase A expressions in MTC patients, both at the gene and protein levels, and to define their prognostic role in MTC assessing their association with lab and clinical parameters.

**Patients and Methods:**

Seventy-one sporadic MTC human samples were analyzed for *RET* mutations and by qPCR for *PTTG1* and *AURKA* (Aurora kinase A) expression. Ki-67 levels and western blot reactivity for PTTG1 and Aurora kinase A were also determined in a selected cohort of patients.

**Results:**

*RET* somatic mutations were found in 48% of the patients (34/71). *PTTG1* expression was statistically different among the groups with or without regional lymph node metastasis (*p* < 0.0001) and advanced stage disease (*p* < 0.01). *PTTG1* and *AURKA* expressions were statistically higher than those of controls (*p* = 0.01 and *p* < 0.002, respectively). *PTTG1* expression and Ki-67 levels were statistically different among the groups with remitted or persistent disease (*p* < 0.05 and *p* < 0.01, respectively). We found a significant correlation between the expressions of *AURKA* and *PTTG1* (*p* < 0.0002, *r* = 0.5298) and between the expressions of *PTTG1* and Ki-67 (*p* = 0.01). Ki-67 levels were statistically different among the groups with or without metastatic lymph nodes (*p* = 0.01) or distant metastases (*p* = 0.003).

**Conclusion:**

The presence of an altered expression of *PTTG1* and *AURKA* is a negative prognostic factor associated with a more aggressive course of disease, such as an advanced stage or disease persistence. It emerges as a cell cycle process mediated by the 2 factors, in addition to the RET pathway, which can be altered in MTC patients.

## 1. Background

Medullary thyroid cancer (MTC) is rare neoplasia that comprises 5-10% of all thyroid tumors [1]. It is characterized by the ability to produce calcitonin, a hormone that regulates the metabolism of calcium and phosphorus. The majority of MTC cases comprise sporadic cases (75%) with unilateral masses that frequently metastasize to the lymph nodes, whereas the hereditary forms (25%) have a genetic basis and may appear as a bilateral or multifocal mass [2]. *RET* (REarranged during Transfection) is an important protooncogene implicated in MTC tumorigenesis. It is mutated in about 50% of the sporadic cases of MTC, and hereditary and sporadic cases show specific mutations which are correlated with phenotype and prognosis [3]. The only potentially curative treatment is surgical resection, though MTC tends to spread at the locoregional area or metastasize at a distance. In these cases, a surgical approach is not always possible and in advanced and progressive MTC cabozantinib and vandetanib can be used [4]. Furthermore, multiple markers show a beneficial value for the diagnosis and prognosis of MTC. Calcitonin and CEA (CarcinoEmbryonic Antigen) are the most significant biochemical markers, in addition to Ca 19.9 (gastrointestinal cancer marker carbohydrate antigen 19.9), while plasma catecholamines, chromogranin A, and urinary markers of catecholamine are the other important ones. Notably, *RET* alteration and Ki-67 value can define patient risk stratification in sporadic MTC [5]. Even if numerous molecular markers have been proposed, no generally accepted indicators can predict the outcome of MTC.

*PTTG1* (Pituitary Tumor-Transforming 1) encodes for a homolog of yeast securin proteins strictly involved in cell cycle regulation, because it is fundamental in hindering separins from promoting sister chromatid separation [6]. It is therefore named human securin, and its involvement in cell transformation and tumorigenesis has been demonstrated [7]. Moreover, it has been found overexpressed in numerous tumors, including endocrine ones, such as pituitary, breast, and ovarian carcinomas. Also, *PTTG1* revealed a pathogenic role in papillary and medullary thyroid cancers, being overexpressed during the metaphase-anaphase transition [8–10]. Also, *AURKA* (Aurora kinase A), a gene that encodes for a serine/threonine kinase needed for G2/M transition, mitosis, and cytokinesis, has been found overexpressed in thyroid cancers and numerous cancer types [11–14]. It must be noted that *AURKA* overexpression or mutation can lead to chromosomal instability, centrosome amplification, and malignant transformation, as a result of cell cycle process deregulation [15].

Given these premises on *PTTG1* and *AURKA* involvement in thyroid tumorigenesis, we decided to explore their association and their prognostic significance in a large cohort of sporadic medullary thyroid cancer samples.

## 2. Patients and Methods

### 2.1. Patients and Biological Specimens

MTC tissues were obtained surgically from 71 patients; 10 thyroid normal (TN) samples were also obtained. All patients underwent total thyroidectomy with the dissection of the regional lymph nodes. The etiology of each thyroid mass, clinical diagnoses, malignancy, and staging were established as previously described [5]. Patients were assumed to have sporadic MTC, as no germline mutation was found, no other endocrine neoplasia was identified, and family history was negative. All studies were performed in accordance to the guidelines of the Declaration of Helsinki. All patients (including those providing normal samples) gave written informed consent to the collection and use of thyroid tissue for research purposes, and the study was approved by the Local Ethics Committee (Azienda Ospedaliera di Padova, code number 12667). After surgery, samples were snap frozen and stored at -80°C.

### 2.2. RET Analysis

DNA was extracted from blood or tissues using the QIAmp DNA Mini Kit (Qiagen, Germany) as per the manufacturer's instruction. DNA was PCR amplified and *RET* (NM_020975.4) exons 5, 8, 10, 11, 13, 14, 15, and 16 were assessed by direct sequencing following previously published procedures [16].

### 2.3. Real-Time PCR (qPCR)

Total RNA was extracted from tissues using the TRIzol reagent lysis buffer (Invitrogen, Carlsbad, CA) from fresh snap-frozen samples according to the manufacturer's protocol. Real-time PCR (qPCR) was performed in an ABI PRISM 7900HT Sequence Detector (Applied Biosystems, Milan, Italy) using the relative quantification method (2^−ΔΔCt^ method) as previously described [17]. Primer sequences for *PTTG1* and *AURKA* were derived from previous works [18, 19]. Data were analyzed with the Sequence Detection Software rel. 2.4 (Applied Biosystems), adopting an automatically set baseline and a fluorescence threshold adjusted to measure quantification cycle (Ct) values. Validation experiments performed using the standard curve method with five serial dilutions of genomic DNA from control subjects showed identical amplification efficiencies (100%±10%) calculated according to the formula *E* = 101/−slope − 1 for all assays. Using the 2^−ΔΔCt^ method, the data were presented as the fold change in gene expression normalized by a reference gene and relative to a calibrator sample. As the reference gene in this study, we used *β*-actin, one of the most commonly used housekeeping genes. A pool of cDNA derived from mixed normal human thyroid tissues was used as the calibrator source in our study.

### 2.4. Ki-67 Immunostaining

Immunohistochemical analysis was performed on 54 paraffin-embedded tissue samples fixed in formalin with 5 mm thick sections, using a standardized avidin-biotin complex method in an automatic system (BenchMark XT; Ventana, Tucson, AZ, USA). The anti-Ki-67 antibody (Rabbit Monoclonal, clone 30-9, Roche) was used. For Ki-67 staining, nuclei were considered positive when a reaction was present as a brown depot (and also when positivity was confined to the nucleoli). Nuclei were negative when no immunostaining was perceived.

A 5 mm^2^ area of the tissue was analyzed for every patient, equal to 3 random 10x fields of view (FOV). A video-analytical method (the Image-Pro Plus 7 analysis program) was used to record the number of positive nuclei in the FOV. The final result was reported as the number of positive nuclei per 1 mm^2^ of the neoplastic area.

### 2.5. Stromal Desmoplasia Evaluation

The presence of a desmoplastic stromal reaction within MTC tissue samples was determined by 2 authors (GP and FG) on 3 *μ*m thick sections from each paraffin-embedded neoplastic tissue sample, stained with ematossilin and eosin. A tumor was considered positive for desmoplasia whenever a collagen-rich stroma as defined above was observed, irrespective of the quantity and extent of this reaction. Tumors with a poor stroma composed only by vessels and sparse reticular fibers were classified as negative for desmoplasia.

### 2.6. Western Blot Analysis

Tumor samples were collected after surgery, frozen immediately in liquid nitrogen, and stored at −80°C. Proteins were extracted and electroblotted onto nitrocellulose membranes as already described [20]. Immunoblot analysis was performed on normal and cancerous MTC tissues. Briefly, membranes were incubated overnight with the following primary antibodies: anti-PTTG1 (GeneTex, Irvine, USA), anti-Aurora kinase A (Cell Signaling Technology, Danvers, USA), and anti-*β*-actin (GeneTex, Irvine, USA). Then, secondary antibodies were added for 1 h with anti-mouse and anti-rabbit (1 : 800) secondary IRDye. Membranes were scanned with the Odyssey CLx system (LI-COR BioSciences, Milan, Italy) equipped with an infrared light technology for the detection. Signal intensity was quantified with Image Studio™ software (Version 4.0, LI-COR BioSciences) following the manufacturer's instructions. The experiments were performed three times.

### 2.7. Statistical Analysis

Statistical analysis was performed using the MedCalc software (version 11.2.1.0) and GraphPad Prism (version 5). The probability with *p* < 0.05 was considered statistically significant. The Kolmogorov-Smirnov test was used to evaluate the normal distribution of each numeric parameter. The comparison between the continuous variables in the considered groups was carried out by means of the *t*-test for independent samples and the Mann–Whitney test. The comparison between categorized variables was performed by the chi-square test or Fisher's exact test. Spearman's correlation analysis was used to determine the possible linearity relationship between the variables under examination.

## 3. Results

### 3.1. Patient Clinical Data

Of all sporadic cases of MTC, 42 were females and 29 were males. The mean age at diagnosis was 61 years (range 35-81 years). The median follow-up was 46 months (range from 6 to 138 months). Tumors were staged according to the TNM classification: 25 were stage I, 16 were stage II, 9 were stage III, and 21 were stage IV (Table 1). It was observed that 29 out of 71 patients were biochemically cured after surgery, while 7 patients were lost during follow-up. In the group with persistent disease (35 patients), 77% (27/35 patients) did not show metastases at diagnosis while 14% (5/35) had metastases at the lymph nodes of the neck. Furthermore, a significantly higher prevalence was observed in biochemically cured women than in men (72% vs. 35%, *p* < 0.01). During the follow-up, 4 patients died due to disease progression.

### 3.2. RET Mutations

No patient had germline mutations in the *RET* oncogene. Genetic *RET* analysis demonstrated a somatic mutation in 48% of patients (34/71) with MTC. 53% of these mutations (18/34) were localized at codon 918 in exon 16 (M918T). 16 mutations were detected in exons 10, 11, and 15. In particular, mutations in exon 10 were 15% (5/34): two in codon 618 (C618S and C618Y), one in codon 513 (A513G), one in codon 609 (C609S), and one in codon 611 (C611R). At exon 11, mutations were 23% (8/34): 18% (6/34) of the cases carried a mutation in codon 634 (C634Y (3), C634W, C634S, and C634R), while 6% (2/34) carried a mutation in codon 630 (C630R). At exon 15, mutations in *RET* were 9% (3/34): 2 patients had the mutation A883F and 1 patient had a 12-base pair deletion starting from codon 898 (p.D898_E901del) (c.2694_2705del12). Of the 4 patients who died, 2 presented the M918T somatic mutation, another the C618S mutation, and the last one the C618Y mutation.

### 3.3. Genotype-Phenotype Correlation

In patients with a mutation, the mean age at diagnosis was lower than that in patients with a wild-type genotype (mean of 58 years, 95% CI 53-63 years versus mean of 64 years, 95% CI 59-69 years, respectively). The prevalence of mutated *RET* was higher in females than in males, 53% versus 47%, respectively. There was no statistically significant association between the presence of somatic *RET* mutation and the size of the tumor or capsular infiltration or the lymph node metastasis at diagnosis or distant metastases.

### 3.4. PTTG1/Securin

**Figure 1:**
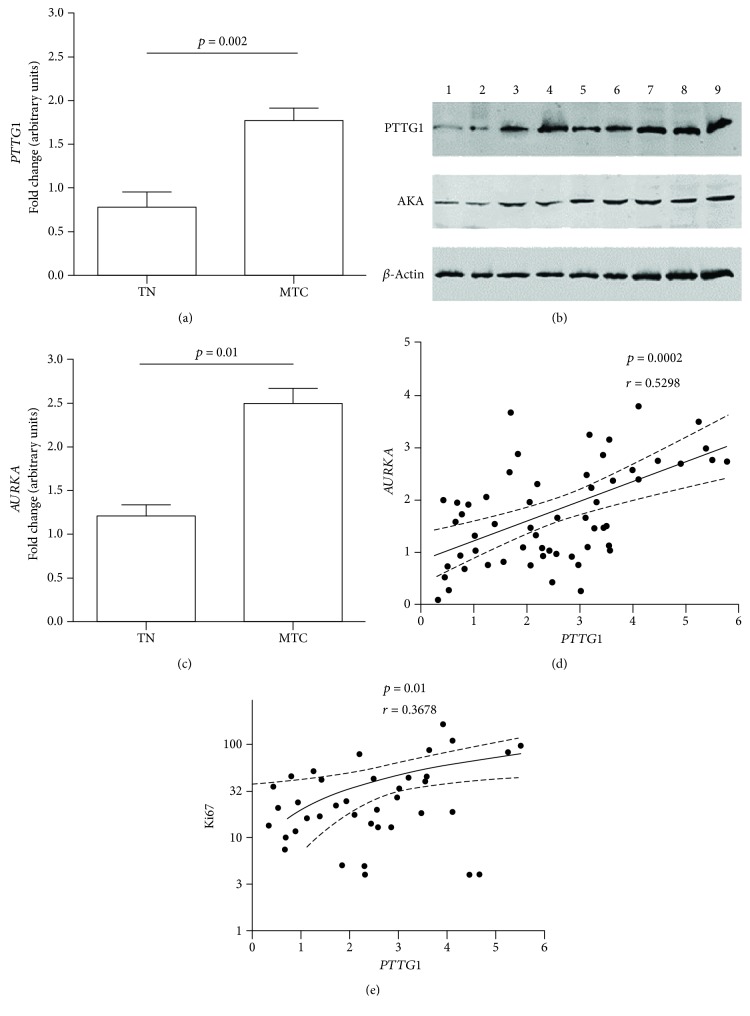
Gene and protein expression levels in MTC patients. (a) *AURKA* (Aurora kinase A gene) expression in 71 MTC patients evaluated by qPCR (TN = normal thyroid). (b) Representative western blot for PTTG1 and AKA (Aurora kinase A) in 6 MTC patients (3: stage I; 4: stage I; 5: stage III; 6: stage IV; 7: stage II; and 8: stage IV), 2 controls (healthy thyroid, numbers 1 and 2), and 1 patient with papillary thyroid carcinoma (number 9). (c) *PTTG1* expression in 71 MTC patients evaluated by qPCR (TN = normal thyroid). (d) Spearman's correlation between *PTTG1* and *AURKA* expression levels for 71 MTC patients evaluated by qPCR. (e) Spearman's correlation between Ki-67 and *PTTG1* expression levels for 54 MTC patients.

The expression of *PTTG1* was evaluated in qPCR in 71 MTC patients. A higher expression of *PTTG1* (approximately 2.5-fold) was shown when compared to the control group (*p* = 0.01) (Figure 1(a)). Furthermore, it was observed that if patients were divided into 2 groups, one in stages I and II and the other in stages III and IV, the 2 groups showed a statistically different *PTTG1* expression (*p* < 0.0001) (Figure S1A). Similarly, if *PTTG1* expression in relation to the value of T (primitive tumor) was divided into 2 groups (one group with T1 and T2 and another with T3 and T4), its expression was statistically different in the 2 groups (*p* < 0.01) (Figure S1B). In addition, *PTTG1* expression was statistically different among the groups with or without regional lymph node metastasis (*p* < 0.0001) (Figure S1C). Also, MTC persistence or remission was evaluated: *PTTG1* expression was statistically different among the groups with remitted or persistent disease (*p* < 0.05) (Figure S1D). No other significant difference was found analyzing for the presence or absence of distant metastases, *RET* mutations, calcitonin value, and tumor size.

We also evaluated the expression of PTTG1 by western blot on 6 MTC patients (a small representative number of patients), 2 controls (healthy thyroid), and 1 patient with papillary thyroid carcinoma (PTC), used as positive control (it has been reported to express elevated quantities of PTTG1 [21]). PTTG1 expression is present in a variable manner in all MTC patients, while healthy controls showed lower reactivity. PTC patients exhibited large amounts of PTTG1 (Figure 1(b)).

### 3.5. AURKA/Aurora Kinase A

*AURKA* expression was evaluated by qPCR in 71 MTC patients. A higher expression of *AURKA* (approximately 1.7-fold) was shown if compared to the control group (*p* < 0.002) (Figure 1(c)). We also observed that the expression levels of *PTTG1* and *AURKA* differ significantly (*p* < 0.01), whereas we found a significant correlation between the expression of *AURKA* and *PTTG1* (*p* < 0.0002) (Figure 1(d)). Several clinical and laboratory parameters were additionally evaluated to discover potential statistical associations, but we did not identify any further significant data.

In addition, we evaluated the expression of Aurora kinase A (AKA) by western blot on 6 MTC patients (a small representative number of patients), 2 controls (healthy thyroid), and 1 patient with papillary thyroid carcinoma (PTC), used as positive control (it has been reported to express elevated quantities of AKA [22]). AKA expression is present in a variable manner in all patients with MTC, while healthy controls showed lower reactivity. PTC patients exhibited large amounts of AKA (Figure 1(b)).

### 3.6. Ki-67 Immunohistochemistry

A statistically different expression emerged in patients with or without metastatic lymph nodes (median 40, 95% CI 19-81/mm^2^ versus 13, 95% CI 7-22/mm^2^, *p* = 0.01) or in those with or without distant metastases (median 40, 95% CI 19-81/mm^2^ to 13, 95% CI 7-22/mm^2^, *p* = 0.003). Also, MTC persistence or remission was evaluated: Ki-67 expression levels were statistically different among the groups with remitted or persistent disease (*p* < 0.01). In addition, we found a significant correlation between the expression levels of Ki-67 and *PTTG1* (*p* = 0.01).

Furthermore, by dividing the expression of Ki-67 into 2 levels (> or <50 cells/mm^2^), the relationship between the disease stage and expression levels could be calculated (*p* < 0.05) (Figure 1(e)). It is notable that the presence of high Ki-67 reactivity is associated with a more advanced stage. Nuclear Ki-67 expression levels were not significantly associated with gender, age, or cancer size.

### 3.7. Stromal Desmoplasia

Desmoplasia was defined as the presence of a newly formed fibrotic collagenous stroma surrounding the invasive neoplastic cells [23]. We evaluated our cohort of samples for the presence or absence of any stromal desmoplasia reaction. We showed a statistically significant association between lymph node metastasis (*p* < 0.001) and stromal desmoplasia (Figure S2A). Similarly, we observed a statistically significant association between *PTTG1* expression (*p* = 0.01) and stromal desmoplasia (Figure S2B).

## 4. Discussion

This work analyzed 71 consecutive sporadic cases of MTC, one of the most representative casuistry from a unique Italian centre. We showed that 48% of patients carried a mutation in the oncogene *RET*, with the M918T alteration the more frequent, as already reported in the literature [3]. Other rare mutations have also been identified and some patients did not show any mutations in the *RET* oncogene, underlining as other genetic *RET* regions can potentially be involved (intronic regions, regulatory regions, etc.) or as other unknown genes may play a specific role in the pathogenesis of MTC. It should be emphasized that the age at diagnosis was lower for mutated patients than wild-type ones: this fact suggests that biomolecular changes in *RET* can induce a more aggressive disease [24]. Correspondingly, higher *PTTG1* expression in MTC patients was associated with advanced stage of disease (stages III and IV) with extracapsular extension (T3 and T4), with lymph node metastases (N1) and persistence of disease, suggesting a potential prognostic value for *PTTG1* in sporadic MTC. The role of PTTG1 protein is two sided: on one hand it regulates the cell cycle and on the other it is involved in tumorigenesis and therefore prooncogenic. In the latter case it has been demonstrated an overexpression (or a non-degradation thereof) in different tumor types [25, 26]. However, the precise role of PTTG1 is not completely elucidated: it is probably cell line dependent and it is implicated in the physiological regulation of the cell cycle even in tumor cells [25]. Nonetheless, a possible implication of PTTG1 in the angiogenic process and in tumor progression has been noted in a follicular thyroid carcinoma model, such as a transgenic TR*β*PV mouse which spontaneously developed this tumor with distant metastases. The deletion of the *pttg1* gene in the transgenic mouse (TR*β* ^PV/PV^ pttg1^−/−^) decreased vascular invasion accompanied by a decrease in FGFR1, FGF-2, and VEGF and decreased tumor mass, although it did not prevent tumor initiation [27]. Whether this can be considered true even in MTC needs to be demonstrated; however, tyrosine kinase inhibitors such as vandetanib and cabozantinib effectively act on VEGFR2 and therefore on VEGF-mediated angiogenesis. It is notable that only the work of Zatelli et al. showed an overexpression of PTTG1 (evaluated by Northern blot) in 19 MTC patients (3 nonsporadic cases) [26]. The authors suggested that this overexpression could be potentially associated with tumor progression, but given the small number of samples, they turned to the cell model to positively substantiate their results. Our results corroborate and reinforce these data, given the wide casuistry analyzed.

Similarly, the expression of *AURKA* in MTC patients was evaluated by a unique work [14]. Baldini and collaborators evaluated *AURKA* in 26 cases of MTC and found no significant correlation with tumor stage (comparable to our data), but we also reported a significant difference between *AURKA* expression levels in MTC when compared to the normal thyroid. This difference could be imputable to the number of samples analyzed, which in our casuistry was larger. Furthermore, AKA is involved in tumorigenesis, being overexpressed in numerous cancer types [22, 28]. Differently from PTTG1, the AKA role in tumor transformation is better known. The upregulation of AKA (but also of Aurora kinase B) induces defects in chromosomal segregation and consequently aneuploidy, which inevitably leads to malignant transformation [29]. In addition, we found an interesting correlation between *AURKA* and *PTTG1*, which relates them to a fundamental function of cell processes: cell cycle control. The 2 factors seem to be associated: a work of Tong et al. demonstrated with a protein array screening that AKA and PTTG1 could be coimmunoprecipitated suggesting an interaction and colocalization of the 2 factors in a cell model of colorectal cancer [30]. The authors also suggested, again by direct demonstration with the *in vitro* model, that the knockdown of *PTTG1* might be a potential approach to improve the efficacy of Aurora kinase inhibitors, drugs not yet approved for cancer treatment, although many clinical trials have been completed or are still ongoing in order to evaluate their use in antitumor therapy.

It is already known that the Ki-67 value is correlated with the mutational status of *RET* and this finding could be useful for the risk stratification of sporadic MTC patients. Moreover, Ki-67 is associated with the presence of metastatic lymph nodes, the presence of metastases, and the persistence of disease [5]. We also observed a correlation between Ki-67 levels and *PTTG1* expression. In the literature, there are only 2 observational works that explored this association. One work showed the significant association between PTTG1 and Ki-67 in pituitary adenomas [31], while the other studied the combination of Ki-67, Galectin-3, and PTTG to distinguish malignant forms of papillary or follicular thyroid cancer [21]. Our finding is therefore the first to associate Ki-67 levels with *PTTG1* expression in sporadic MTC. We can speculate that these 2 factors can play a substantial role in tumor cell processes: while PTTG1 is involved in the cell cycle, Ki-67 is a marker of active cell proliferation, altered in numerous neoplasms and associated with the prognosis [32]. However, the statistical association does not imply a causal relationship, and indeed, there are no works that can demonstrate a direct interaction of PTTG1 and Ki-67 from a biomolecular point of view, unlike for *PTTG1* and *AURKA*.

Furthermore, the presence of stromal desmoplasia was investigated in MTC samples by paraffin-embedded tissue sections. We showed a strong correlation of stromal desmoplasia with lymph node metastasis (*p* < 0.001) (Figure S2A). This result is in agreement with previous findings [23] and confirms the prognostic potential of this data, as also suggested by *PTTG1* expression, which is strictly associated (*p* = 0.01) with stromal desmoplasia (Figure S2B).

In conclusion, in sporadic MTC, the presence of an altered expression of *PTTG1* and *AURKA* is a negative prognostic factor associated with a more aggressive course of disease (advanced stage and disease persistence). However, the presence of progressive disease and the risk of death also depend on other molecular mechanisms acting on neoplastic proliferation, of which the expression of Ki-67 is a possible index. In addition to PIK3/Akt and MAPK pathways activated by RET, it is possible that PTTG1 and AKA could be implicated in further intracellular signaling pathways not yet sufficiently investigated, in particular those related to the cell cycle process. More work is needed to elucidate this hypothesis.

## Figures and Tables

**Table 1:** Clinical and histological features and follow-up in patients with sporadic MTC in relation to stages I-II and stages III-IV.

Tumor stage	Stages I-II (*n* = 41)	Stages III-IV (*n* = 30)
Age at diagnosis	55 (95% CI 49-62)	61 (95% CI 55-67)
Gender (F/M)	30/11	10/20
Tumor category (cancer staging)		
T1 (27)	23	4
T2 (13)	9	4
T3 (27)	9	18
T4 (4)	0	4
Capsular infiltration	5	18
Lymph node metastasis		
N- (43)	41	2
N+ (28)	0	28
Distant metastasis		
M- (63)	41	22
M+ (8)	0	8
RET mutation		
*RET-* (37)	22	15
*RET+* (34)	19	15
*Outcome*		
Free from disease (29)	27	2
Persistent disease (35)	7	28
Survival		
Dead	0	4
Live	41	26

CI: confidence interval.

## Data Availability

Data sharing is not applicable to this article as no datasets were generated or analysed during the current study.

## Supplementary Materials

Supplementary Materials Figure S1: PTTG1 expression levels by qRT-PCR analysis. (a) Relationship between PTTG1 levels and the stage. (b) Relationship between PTTG1 levels and the value of T. (c) Relationship between PTTG1 levels and the presence (N+) or absence (N-) of regional lymph node metastasis. (d) Relationship between PTTG1 levels and remitted or persistent disease. Each circle represents an individual sample; each bar represents the mean value of PTTG1; vertical lines represent 95% CI. Figure S2: stromal desmoplasia statistical analysis. (a) Association between stromal desmoplasia and lymph node metastasis (Fisher's exact test): *n* = 0, no lymph node metastasis; *n* = 1, lymph node metastasis. (b) Association between stromal desmoplasia and mean PTTG1 expression by box plot representation (independent sample *t*-test).
