# Supplementary material for: Novel Prognostic Factors Associated with Cell Cycle Control in Sporadic Medullary Thyroid Cancer Patients
Source: Int J Endocrinol. 2019 Feb 18;2019:9421079. doi: 10.1155/2019/9421079 (PMC6398011; doi:10.1155/2019/9421079)

**Supplementary figures legend**

**Figure S1.**

*PTTG1* expression levels by qRT-PCR analysis. A) Relationship between *PTTG1* levels and the stage. B) Relationship between *PTTG1* levels the value of T. C) Relationship between *PTTG1* levels and the presence (N+) or absence (N-) of regional lymph nodes metastasis. D) Relationship between *PTTG1* levels and remitted or persistent disease. Each circle represents an individual sample; each bar represents the mean value of *PTTG1*, vertical lines 95% CI.


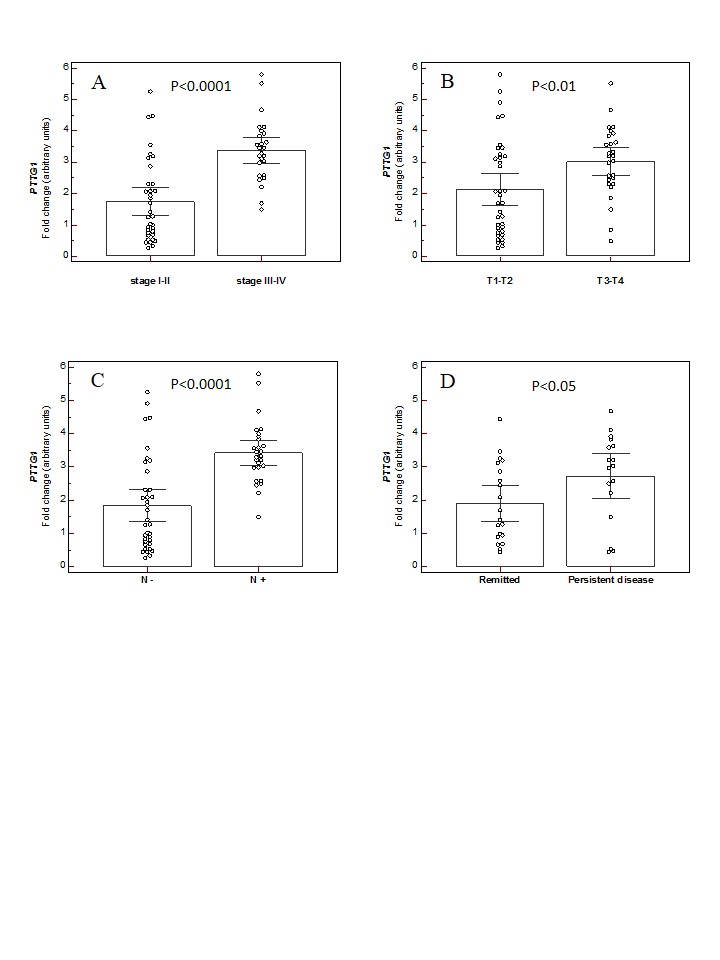


**Figure S2.**

Stromal desmoplasia statistical analysis. A) association between stromal desmoplasia and lymph node metastasis (Fisher’s exact test); N=0 no lymph node metastasis, N=1 lymph node metastasis. B) association between stromal desmoplasia and mean *PTTG1* expression by box plot representation (independent samples t-test).


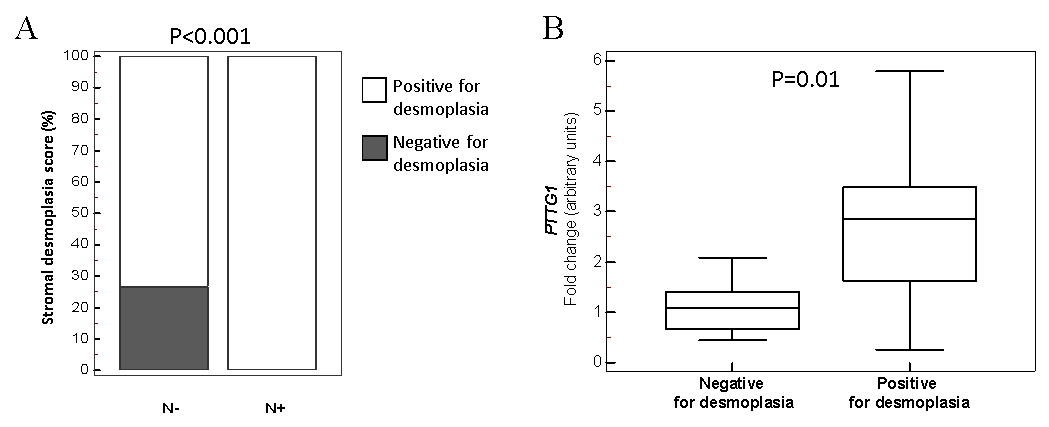

Supplement: Supplementary Materials — Figure S1: PTTG1 expression levels by qRT-PCR analysis. (a) Relationship between PTTG1 levels and the stage. (b) Relationship between PTTG1 levels and the value of T. (c) Relationship between PTTG1 levels and the presence (N+) or absence (N-) of regional lymph node metastasis. (d) Relationship between PTTG1 levels and remitted or persistent disease. Each circle represents an individual sample; each bar represents the mean value of PTTG1; vertical lines represent 95% CI. Figure S2: stromal desmoplasia statistical analysis. (a) Association between stromal desmoplasia and lymph node metastasis (Fisher's exact test): n = 0, no lymph node metastasis; n = 1, lymph node metastasis. (b) Association between stromal desmoplasia and mean PTTG1 expression by box plot representation (independent sample t-test). [file 9421079.f1.docx]
